# Massachusetts

**Published:** 1895-05

**Authors:** 


### MASSACHUSETTS.

The Veterinary Registration bill now pending before the Legislature of Massachusetts is reported in good position, and will no doubt become a law of the State. It will be a good stepping-stone toward better regulations.

The bill known as the Tuberculosis bill in the Legislature of Massachusetts has been altered in the Senate by the adoption of Senator Bill's amendment, which provides for the use of tuberculin only where the owner requests it, and where the disease is known to exist after a physical examination.

We feel sure that the wide difference of opinion existing among the veterinary profession of Massachusetts has not arisen so much from a real disagreement of views as to the value of certain methods of diagnosis of tuberculosis as to the fact that the time and people were not opportune for pursuing the subject on such a broad and thorough plan.

The bill which passed the Senate of Massachusetts recently reduced the proposed appropriation to the State Tuberculosis Commission from $212,000 to $100,000, and proposes to allow full compensation where animals are destroyed. It is now in the hands of the House.

